# Adenylyl cyclase 4 does not regulate collecting duct water and sodium handling

**DOI:** 10.1002/phy2.277

**Published:** 2014-03-26

**Authors:** Wararat Kittikulsuth, Deborah Stuart, Donald E. Kohan

**Affiliations:** 1 Division of Nephrology, University of Utah Health Sciences Center, Salt Lake City Veterans Affairs Medical Center, Salt Lake City, Utah

**Keywords:** Adenylyl cyclase 4, collecting duct, sodium excretion, vasopressin, water excretion

## Abstract

Adenylyl cyclase (AC)‐stimulated cAMP is a key mediator of collecting duct (CD) Na and water transport. AC isoforms 3, 4, and 6 are expressed in the CD. Our group demonstrated that AC6, but not AC3, is involved in regulating CD Na and water transport. However, the role of AC4 in such regulation remains unknown. Therefore, we generated mice with loxP‐flanked critical exons in the *Adcy4* gene and bred with mice expressing the aquaporin‐2/Cre recombinase transgene to yield CD principal cell‐specific knockout of AC4 (CD AC4 KO). Isolated inner medullary CD showed 100% genomic target gene recombination in CD AC4 KO mice, while microdissected cortical CD and renal papillary AC4 mRNA was significantly reduced in CD AC4 KO mice. CD AC4 KO had no effect on vasopressin (AVP)‐stimulated cAMP generation in the inner medulla. Water intake, urine volume, and urine osmolality were similar between CD AC4 KO and control mice during normal or restricted water intake. Sodium intake, urinary Na excretion, and blood pressure on a normal‐, high‐, or low‐Na diet were not affected by CD AC4 KO. Moreover, there were no differences in plasma AVP or plasma renin concentration between CD AC4 KO and control mice. In summary, these data suggest that CD AC4 does not play a role in the physiologic regulation of CD Na and water handling.

## Abstract (precis)

Principal cells in the collecting duct express adenylyl cyclase 4 (AC4), however, the role of AC4 in the regulation of collecting duct function is unknown. We made mice with collecting duct principal cell‐specific deletion of AC4 and found that these mice have no alterations in arterial pressure or urinary sodium, potassium, or water excretion under varying physiological conditions.

## Introduction

Adenylyl cyclases (ACs) are a family of enzymes that catalyze the synthesis of cAMP from ATP; cAMP is a secondary messenger for a wide variety of hormones modulating renal function, including vasopressin (AVP) (Schafer and Troutman 1990; Wallace et al. 2002), angiotensin II (Ang II) (Lee et al. 2007; Li et al. 2011), and prostaglandin E_2_ (Nasrallah et al. 2001). To date, nine membrane‐bound AC isoforms and one soluble AC have been identified (Beazely and Watts 2006); of the membrane‐bound ones, our group has demonstrated that AC3, AC4, and AC6 are expressed throughout the collecting duct (CD) (Strait et al. 2010). Mice with global AC6 deficiency have decreased urine osmolality, increased urine output, and increased fluid intake (Chien et al. 2010; Rieg et al. 2010). Furthermore, mice lacking AC6 in principal cells of the CD (CD AC6 KO) show mild urinary concentrating defects (Roos et al. 2012) as well as abolished AVP‐stimulated epithelial Na channel (ENaC) activity (Roos et al. 2013). With regard to AC3, global AC3 KO tends to increase urine volume and urinary Na excretion, while glomerular filtration rate is reduced by 50% (Pluznick et al. 2009). Recently, we demonstrated that CD‐specific KO of AC3 in mice has no effect on renal water and sodium handling, and no alteration in AVP‐stimulated cAMP accumulation or ENaC activity (Kittikulsuth et al. 2014). While the physiological roles of AC3 and AC6 in regulating AVP action in the CD have now been examined, the role of the remaining identified CD membrane‐bound AC isoform, AC4, remains largely unknown. One previous study by our group found that small interfering RNA (siRNA) against AC3 and AC6, not AC4, in inner medullary CD (IMCD) cells blunted AVP‐stimulated cAMP production (Strait et al. 2010), however, no relevant in vivo studies have been conducted. Consequently, the goal of the present study was to investigate the effect of CD‐specific KO of AC4 on renal Na and water handling.

## Material and Methods

### Animal study approval

All animal use and welfare adhered to the NIH Guide for the Care and Use of Laboratory Animals. Animal breeding, housing, and protocols were approved by the Institutional Laboratory Animal Care and Use Committee of the University of Utah Health Sciences Center.

### Generation of CD AC4 KO

Floxed (loxP‐flanked) AC4 mice were generated with loxP sites flanking exons 3–7 of the *Adcy4* gene, the exons encoding part of the transmembrane, and the first catalytic domains. In brief, a targeting construct was made containing loxP sites in introns 3 and 7 of the *Adcy4* gene with ~2.9 kb flanking homology arms. A FRT‐flanked neomycin resistance cassette was inserted immediately 3′ to the loxP site in intron 3. Mice were generated using homologous recombination in embryonic stem cells, blastocyst injection, and identification of founders conferring germline transmission of the floxed allele. The neomycin resistance cassette was eliminated by breeding with mice expressing Flp recombinase under control of the ROSA26 promoter (Meyers et al. 1998). The Flp recombinase was then bred out of floxed AC4 mice by crossing with wild‐type mice. To generate CD‐specific deletion of the *Adcy4* gene, floxed AC4 mice were bred with aquaporin‐2 (AQP2)‐Cre mice, which contain a transgene with 11 kb of the mouse AQP2 gene 5′ flanking region driving expression of Cre recombinase (Nelson et al. 1998). Female AQP2‐Cre mice were mated with male floxed AC4 mice; female offspring heterozygous for floxed AC4 and hemizygous for AQP2‐Cre were bred with males homozygous for floxed AC4. Animals homozygous for floxed AC4 and hemizygous for AQP2‐Cre (CD AC4 KO) were used in all studies. Gender‐matched littermates that were homozygous for the floxed *Adcy4* gene, but without Cre, were used as controls in all studies.

### Genotyping

Tail DNA from floxed control mice was PCR amplified with the following primers: AC4F 5′‐ ccctgtttccttgtgtatgg‐3′ and AC4R 5′‐agatatctgaagccaggctg‐3′, which yields a 353 bp product from the floxed *Adcy4* gene and a 319 bp product from the wild‐type allele. The AQP2‐Cre transgene was detected by using primers mAQP2F 5′‐gagacgtcaatccttatctggag‐3′, creTagR 5′‐gcgaacatcttcaggttctgcgg‐3′, and R_2_D_2_R 5′‐ggctactcacagcattgacagc‐3′, which yield 600 and 650 bp products for AQP2‐Cre and wild‐type DNA, respectively.

### Analysis of Adcy4 gene recombination

Brain, heart, lung, spleen, liver, intestine, testis, and kidneys were excised. Kidneys were cut longitudinally into sections containing the entire corticomedullary axis. The kidney sections were incubated with 1 mL of Hanks Balance Salt Solution (HBSS) containing 2 mg/mL collagenase and 2 mg/mL hyaluronidase for 20 min at 37°C. The incubated tissue was rinsed with HBSS and stored on ice until dissection of the tubules. Dissection of proximal tubules and cortical and inner medullary CDs was performed at 4°C. DNA from selected organs and microdissected tubules was isolated and PCR amplified to evaluate for *Adcy4* gene recombination using primers spanning exons 3–7 of the *Adcy4* gene: F 5′‐ctcaaaggtatgagttctcatc‐3′ and R 5′‐gagatctctgcacagatgtg‐3′. Recombination of the *Adcy4* gene yields a 250 bp product; the size of the unrecombined *Adcy4* gene is ~2900 bp.

### Water and salt studies

CD AC4 KO and their floxed controls were placed in metabolic cages and given 9 mL of a gelled diet made from 62 g of PMI rodent powdered diet (LD101; LabDiet, Richmond, IN), 7 g gelatin and 110 mL water (Ahn et al. 2004) with free access to drinking water for 3 days (baseline). The mice do not consume all the gel, that is, they are not forcibly limited to eat a fixed amount of food. For moderate water restriction, mice were switched to 9 mL of gelled diet containing 124 g of PMI rodent powdered diet, 7 g gelatin and 110 mL water with free access to drinking water for 3 days. We have empirically found that increasing the powdered diet concentration in the gel reduces water intake, even when the mice are given free access to drinking water. For marked water restriction, mice were given 9 mL gelled diet containing 248 g of PMI rodent powdered diet, 7 g gelatin and 110 mL water for 2 days with no access to drinking water. Urine was analyzed for volume and osmolality.

For Na balance studies, mice were fed a normal (0.3%) Na diet for 3 days followed by a high (3.15%) Na diet for 7 days and then a low (0.03%) Na diet for 5 days. The diets consisted of the normal water gelled diet above with NaCl modified to achieve the low or high‐Na diets. At the end of each diet, blood was taken from the tail vein for determination of plasma renin concentration (PRC). A 24‐h urine collection was done every day. Urines from days 2 and 3 of a normal‐Na diet and for days 2, 3, and 5 of high‐or low‐Na diets were analyzed for volume, Na and K.

### Blood pressure monitoring

Blood pressure was monitored in CD AC4 KO and their floxed controls by radiotelemetry (TA11‐PAC10; Data Sciences International, St. Paul, MN) with catheters inserted into the right carotid artery. The mice were allowed to recover for 1 week after surgery. Blood pressure and heart rate were monitored during normal‐, high‐ and low‐Na intake.

### AVP‐stimulated cyclic AMP production

Inner medullas were isolated, minced in Hank's Balanced Salt Solution (HBSS) and centrifuged to bring down intact tubules and cells. The pellet was resuspended in HBSS + 10 mmol/L HEPES, pH 7.4 and incubated with 1 mmol/L 3‐isobutyl‐1‐methylxanthine for 15 min at 37°C followed by AVP (1 nmol/L–1 *μ*mol/L, Sigma, St. Louis, MO) or 100 nmol/L angiotensin II (Ang II, Sigma) for 10 min. Cells were extracted with 100% ethanol and cAMP levels measured by enzyme immunoassay (Enzo Life Sciences, Farmingdale, NY). Protein concentration was determined by the Bradford assay (Bio‐Rad, Hercules, CA).

### Analysis of mRNA

RNA from microdissected cortical CD (CCD) and renal papilla of CD AC4 KO and floxed controls was extracted using ZR RNA MicroPrep™ (Zymo, Irvine, CA) and RNeasy Mini Kit (Qiagen, Valencia, CA), respectively. The reverse transcription process was performed using Omniscript RT Kit (Qiagen). cDNA levels were determined for AC3, AC4, AC6, and GAPDH using real‐time PCR (StepOne Plus; Applied Biosystems, Foster City, CA). PCR was performed according to instructions provided by the manufacturer using the *Taq*man Gene Expression Assay (Applied Biosystems) with *Adcy3* (Cat.# Mm00460371_m1), *Adcy4* (Cat.# Mm00475491_m1), *Adcy6* (Cat.# Mm00475773_g1) and GAPDH (Cat.# Mm99999915_g1) primers.

### Hormone and electrolyte analysis

Urine Na and K concentration were analyzed using an Easylyte Analyzer (Medica, Bedford, MA). Urine osmolality was determined by freezing point depression (Osmett II; Precision System, Natick, MA). For plasma AVP analysis, mice were decapitated, blood collected, plasma extracted using acetone and petroleum ether, and AVP determined by enzyme immunoassay (Enzo Life Sciences). Plasma renin concentration was measured by enzyme immunoassay as the amount of angiotensin I (Ang I) generated after incubation with excess angiotensinogen (Peninsula Laboratories, San Carlos, CA) and expressed as the amount of Ang I generated per hour per mL of plasma.

### Statistical analysis and data presentation

Data are reported as mean ± SEM. Studies involving varying Na and water intakes and hemodynamics were analyzed by analysis of variance. Plasma AVP, PRC, mRNA, and cAMP studies were analyzed by a two‐sided unpaired Student's *t*‐test except where indicated otherwise. The criterion for significance was *P *≤**0.05.

## Results

### Confirmation of CD AC4 KO mice

**Figure 1.**
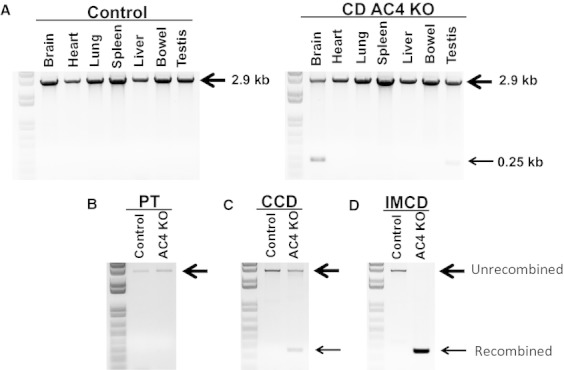
Recombination of the *Adcy4* gene DNA in floxed control and CD AC4 KO mice from: (A) organ panels; (B) proximal tubule; (C) cortical collecting duct; and (D) inner medullary collecting duct. The upper 2.9 kb band is the unrecombined allele (large arrowhead in bottom panels) and the 0.25 kb band (small arrowhead in bottom panels) is the recombined allele.

CD AC4 KO mice were born at the expected frequency, had normal growth rates (as determined by body weight) and no apparent gross abnormalities. Kidneys had normal gross morphology and histology by light microscopy. To examine the degree of *Adcy4* gene recombination, DNA from multiple organs was analyzed. DNA from heart, lung, spleen, liver, and bowel showed no recombination of the *Adcy4* gene. However, there was some degree of recombination in brain and testis in CD AC4 KO mice (Fig. 1A), as is typically seen with the AQP2‐Cre transgene (Kittikulsuth et al. 2014). Analysis of DNA from microdissected tubules from CD AC4 KO mice revealed no *Adcy4* gene recombination in proximal tubules (Fig. 1B), some degree of *Adcy4* gene recombination in CCD (contains untargeted intercalated cells) (Fig. 1C) and complete recombination in IMCD (Fig. 1D).

**Figure 2.**
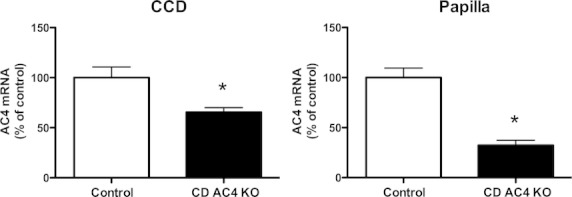
Relative mRNA expression of AC4/GAPDH in microdissected CCD (A) and renal papilla (B) from CD AC4 KO and floxed control mice. *P < 0.05 versus floxed control (*n* = 5–6 mice/group).

We further determined AC4 message in microdissected CCD and renal papilla from CD AC4 KO and control mice. Note that renal papilla was studied instead of microdissected IMCD as we have found that the process of microdissecting IMCD largely destroys RNA (but not DNA), possibly due to the very sticky nature of IMCD and associated tissue (CCD, by comparison, are not sticky and are readily microdissected and analyzed for RNA). Real‐time PCR of CCD and renal papilla from CD AC4 KO mice showed 35 and 65% reductions of AC4 mRNA, respectively, as compared with controls (Fig. 2A and B). As AC3 and AC6 are expressed in CD, and at least AC6 is involved in cAMP‐regulated CD water and Na transport (Strait et al. 2010; Roos et al. 2012, 2013; Kittikulsuth et al. 2014), we further examined AC3 and AC6 mRNA in CCD and renal papilla. There were no differences in AC3 and AC6 mRNA in CCD between CD AC4 KO and control mice (AC3: 1.0 ± 0.2 for control vs. 0.8 ± 0.3 for CD AC4 KO; AC6: 1.0 ± 0.2 for control vs. 1.0 ± 0.3 for CD AC4 KO; *n* = 5–6 mice/group, units are relative value compared to control). Furthermore, the renal papilla from CD AC4 KO and control mice expressed a comparable level of AC3 or AC6 mRNA (AC3: 1.0 ± 0.2 for control vs. 0.8 ± 0.1 for CD AC4 KO; AC6: 1.0 ± 0.1 for control vs. 1.1 ± 0.1 for CD AC4 KO; *n* = 5–6 mice/group). Taken together with the DNA recombination data, within our ability to evaluate the degree of KO, these findings suggest that the *Adcy4* gene is effectively targeted selectively in CD principal cells within the kidney.

### Vasopressin‐stimulated cAMP production from minced IM

**Figure 3.**
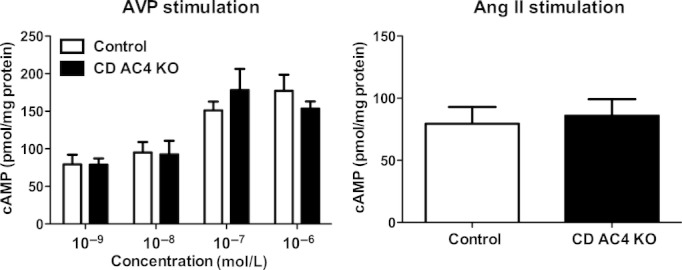
Cyclic AMP production in minced inner medulla from CD AC4 KO and floxed controls after vasopressin (AVP; A) or angiotensin II (AngII; B) stimulation. Samples were stimulated with 1 nmol/L–1 *μ*mol/L AVP or 100 nmol/L AngII for 10 min in the presence of 1 mmol/L IBMX (*n* = 8–17 mice/group).

The role of CD AC4 in mediating AVP‐stimulated cAMP production was examined. AVP‐stimulated cAMP generation from minced inner medulla in a dose‐dependent manner in both CD AC4 KO and control mice (Fig. 3A). However, there was no difference in AVP‐stimulated cAMP production between the two groups at any concentrations of AVP (Fig. 3A). We further determined cAMP generation using Ang II as it was previously demonstrated that Ang II‐stimulated cAMP production is reduced in CD AC3 KO mice (Kittikulsuth et al. 2014). However, Ang II‐increased cAMP production in minced inner medulla in CD AC4 KO and control mice to a similar extent (Fig. 3B). Please note that vehicle‐treated inner medulla typically has nondetectable cAMP, hence values for vehicle treatment alone are not shown.

### Effect of CD AC4 KO on water handling

CD AC4 KO and control mice were placed on a normal water intake (~5 mL/day), moderate water intake (~4 mL/day), and severe water restriction (~1.5 mL/day). Table 1 shows that no differences occurred in water intake between the two groups with any levels of water intakes. Similarly, urine flow, urine osmolality, and urinary osmolyte excretion were not different between CD AC4 KO and control mice regardless of the degree of water intake.

**Table 1.** Effect of CD AC4 KO on fluid intake, urine volume, and urine osmolality during normal, moderate, and marked water restriction (*n* = 13–14 mice/group).

	Control	CD AC4 KO
Normal water intake		
Fluid intake, mL	5.0 ± 0.2	4.9 ± 0.3
Urine flow, mL	2.6 ± 0.1	2.4 ± 0.1
Urine osmolality, mosmol/kgH_2_O	2555 ± 67	2594 ± 133
Osmolyte excretion, mosmol/day	6.6 ± 0.2	6.0 ± 0.3
Moderate water restriction		
Fluid intake, mL	4.2 ± 0.4	3.9 ± 0.2
Urine flow, mL	1.9 ± 0.2	1.5 ± 0.1
Urine osmolality, mosmol/kgH_2_O	2787 ± 204	3065 ± 139
Osmolyte excretion, mosmol/day	4.9 ± 0.4	4.5 ± 0.3
Marked water restriction		
Fluid intake, mL	1.6 ± 0.1	1.5 ± 0.1
Urine flow, mL	0.8 ± 0.1	0.9 ± 0.1
Urine osmolality, mosmol/kgH_2_O	4539 ± 180	4236 ± 127
Osmolyte excretion, mosmol/day	3.6 ± 0.3	3.6 ± 0.3

### Effect of CD AC4 KO on blood pressure and sodium handling

**Figure 4.**
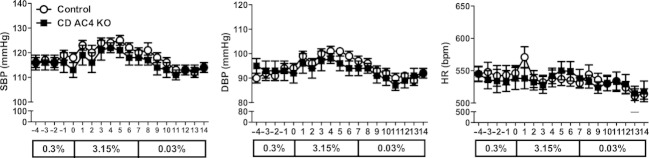
Effect of CD AC4 KO on systolic (A) and diastolic (B) blood pressure, and heart rate (C) during normal‐ (0.3% Na), high‐ (3.15% Na), and low‐ (0.03% Na) salt intake (*n* = 11–14 mice/group).

Telemetric blood pressure (BP) and heart rate were determined in CD AC4 KO and control mice during 5 days of normal Na intake (0.3% Na), followed by 7 days of high‐Na intake (3.15% Na), and then 7 days of low‐Na intake (0.03% Na). There were no differences in systolic BP (Fig. 4A), diastolic BP (Fig. 4B), or heart rate (Fig. 4C) between CD AC4 KO and the controls on any of the days during normal‐, high‐ or low‐Na feeding.

After completion of the telemetric analysis, CD AC4 KO and control mice were placed on a normal‐Na diet for 3 days (baseline), switched to a high‐Na diet for 7 days, followed by a low‐Na diet for another 5 days. Food and water intakes were similar between both groups on a normal‐Na diet. There were no differences in urine flow, urine concentration of Na and K, as well as urinary excretion of Na and K during normal‐Na feeding between CD AC4 KO and control mice (Table 2). Table 3 shows metabolic balance data from day 2, day 3, and day 5 of a high‐ or low‐Na diet. The amount of food intake was significantly higher in CD AC4 KO mice as compared to controls on day 2 of high‐Na intake, but it became similar between the two groups on day 3 and day 5. Urine Na concentration was higher in CD AC4 KO mice on day 3 of high‐salt feeding; however, there was no difference in this parameter on day 2 and day 5 of a high‐salt diet. Furthermore, fluid intake, urine K concentration, and urinary excretion of Na or K were comparable between the two groups on all days of high‐salt feeding. During low‐salt feeding, there were no differences in food intake, water intake, urine volume, urinary Na or K concentration, or urine Na or K excretion between the two genotypes.

**Table 2.** Baseline metabolic balance characteristics on days 2 and 3 of a normal‐Na intake with free access to water (*n* = 13–14 mice/group).

	Day 2		Day 3	
	Control	CD AC4 KO	Control	CD AC4 KO
Normal‐salt intake				
Food intake, g	8.0 ± 0.3	7.6 ± 0.3	7.8 ± 0.3	7.5 ± 0.3
Fluid intake, mL	5.6 ± 0.3	5.2 ± 0.4	5.0 ± 0.2	4.9 ± 0.3
Urine flow, mL	2.7 ± 0.1	2.5 ± 0.2	2.6 ± 0.1	2.4 ± 0.1
Urine [Na], mmol/L	112 ± 5	115 ± 5	120 ± 3	114 ± 4
Urine [K], mmol/L	118 ± 5	127 ± 5	119 ± 3	121 ± 4
UNaV, *μ*mol/day	299 ± 17	282 ± 19	312 ± 14	272 ± 18
UKV, *μ*mol/day	315 ± 18	308 ± 17	310 ± 13	282 ± 13

UNaV, urinary Na excretion; UKV, urinary K excretion.

**Table 3.** Effect of CD AC4 KO on Na and K balance on days 2, day 3, and day 5 after initiation of high‐ or low‐salt intake with free access to water (*n* = 13–14 mice/group).

	Day 2		Day 3		Day 5	
	Control	CD AC4 KO	Control	CD AC4 KO	Control	CD AC4 KO
High‐salt intake						
Food intake, g	5.1 ± 0.2	5.9 ± 0.4*	5.5 ± 0.2	6.0 ± 0.4	5.2 ± 0.2	5.5 ± 0.3
Fluid intake, mL	8.1 ± 0.4	8.3 ± 0.5	8.3 ± 0.5	8.4 ± 0.7	8.8 ± 0.5	8.8 ± 0.6
Urine flow, mL	5.3 ± 0.3	5.6 ± 0.4	5.5 ± 0.4	5.5 ± 0.4	5.8 ± 0.4	6.1 ± 0.5
Urine [Na], mmol/L	415 ± 22	430 ± 16	413 ± 22	477 ± 16*	425 ± 20	457 ± 13
Urine [K], mmol/L	61 ± 3	64 ± 2	63 ± 3	72 ± 3	61 ± 2	62 ± 2
UNaV, *μ*mol/day	2157 ± 137	2371 ± 157	2218 ± 120	2624 ± 235	2408 ± 126	2778 ± 193
UKV, *μ*mol/day	314 ± 17	347 ± 17	337 ± 16	393 ± 28	346 ± 16	374 ± 22
Low‐salt intake						
Food intake, g	7.5 ± 0.3	8.3 ± 0.8	7.6 ± 0.4	7.4 ± 0.4	7.5 ± 0.3	6.7 ± 0.4
Fluid intake, mL	5.0 ± 0.2	5.6 ± 0.5	5.2 ± 0.3	5.7 ± 0.5	5.2 ± 0.3	4.8 ± 0.2
Urine flow, mL	2.9 ± 0.2	2.7 ± 0.2	2.6 ± 0.2	2.7 ± 0.2	2.9 ± 0.2	2.6 ± 0.2
Urine [Na], mmol/L	18 ± 1	19 ± 1	16 ± 1	15 ± 1	15 ± 1	14 ± 1
Urine [K], mmol/L	96 ± 5	101 ± 6	90 ± 5	97 ± 3	107 ± 5	103 ± 4
UNaV, *μ*mol/day	52 ± 4	50 ± 4	43 ± 4	40 ± 2	43 ± 3	38 ± 3
UKV, *μ*mol/day	276 ± 22	271 ± 20	237 ± 19	263 ± 20	310 ± 22	266 ± 22

UNaV, urinary Na excretion; UKV, urinary K excretion.

* *P* < 0.05 versus floxed control on the same day and similar diet.

### Effect of CD AC4 on plasma hormones

In order to detect any alterations in key regulators of renal Na and water excretion, possibly in compensation for changes induced by CD AC4 KO, the levels of plasma AVP and plasma renin concentration (PRC) in CD AC4 KO and control mice were determined. There were no differences in plasma AVP between the two groups when fed a normal or low‐water diet, nor were there any differences in PRC between the two groups when fed a normal or low‐salt diet (Table 4).

**Table 4.** Effect of CD AC4 KO on plasma AVP and plasma renin concentration (PRC) during normal‐ and low‐salt or water intake (*n* = 8–13 mice/group).

	Control	CD AC4 KO
Plasma AVP, pg/mL		
Normal‐water intake	24 ± 8	30 ± 14
Low‐water intake	90 ± 14	62 ± 14
PRC, ng/min/h		
Normal‐salt intake	77 ± 7	71 ± 7
Low‐salt intake	147 ± 32	103 ± 21

## Discussion

The current study demonstrates that CD‐specific KO of AC4 in mice has no effect on: (1) urine volume and urine osmolality during different levels of water intake; (2) urinary Na and K excretion or BP during a normal‐, high‐, or low‐Na diet; (3) cAMP generation by the inner medulla after AVP or Ang II incubation; and (4) plasma AVP or PRC. These data support the notion that CD AC4 is not responsible for AVP‐stimulated cAMP production or cAMP‐mediated actions on water and Na handling in the CD. Notably, these results are in agreement with previous in vitro findings wherein siRNA knockdown of AC4 in cultured mouse IMCD did not alter AVP‐stimulated cAMP production (Strait et al. 2010).

Adenylyl cyclase 4, as well as AC2 and AC7, are classified as group II ACs, which are unique by virtue of their ability to be regulated by all members of the G protein family, including *βγ*‐subunits (G*βγ*) (Defer et al. 2000; Linder 2006; Willoughby and Cooper 2007; Sadana and Dessauer 2009). AC4 is widely distributed throughout the body, including brain, eyes, heart, lung, adipose tissue, ovary, adrenal gland, liver, and kidney (Gao and Gilman 1991; Zhang et al. 2000; Serazin‐Leroy et al. 2001; Rui et al. 2004; Bogard et al. 2011; Bagavandoss and Grimshaw 2012). On a subcellular level, AC4 resides in the bulk plasma membrane, whereas AC3 and AC6 are associated with lipid rafts (Cooper and Crossthwaite 2006). While speculative, the possibility exists that such membrane compartmentalization explains, at least in part, our current findings in that AC6 may be associated in the lipid raft with V2 receptors, while AC4 may be associated with other receptors that lie outside caveolae, such as EP2 or EP4 receptors (Ostrom et al. 2001; Bogard et al. 2011). Ultimately, studies examining the role of AC4 in mediating prostaglandin as well as other agonist‐induced cAMP accumulation in the CD will be needed.

While the tissue distribution of AC4 has been well characterized, very few studies have examined the biologic actions of AC4. Unlike the other class II AC isoforms, AC4 is inhibited by protein kinase C (Zimmermann and Taussig 1996; Defer et al. 2000; Linder 2006; Sadana and Dessauer 2009). Al‐Hakim et al. (2004) demonstrated that AC4 was involved in cAMP‐induced cell proliferation in cultured adrenal cells. El‐Haroun et al. (2004) reported that inflammatory cytokines reduced prostanoid‐induced cAMP production by downregulation of AC1, AC2 and AC4 in human pulmonary artery smooth muscle cells. Wang and Burns (2006) showed that AC2 or AC4 were stimulated by G*βγ* and were involved in opioid tolerance. Taken together, it is evident that AC4 is likely to serve some unique function, however, due to the rarity of studies investigating this AC isoform and the lack of any in vivo analysis, the biologic roles of AC4 are essentially unknown. To our knowledge, the present study is the first to examine the biologic actions of AC4 in vivo. Clearly, further analysis is needed, including generation of mice with whole body disruption of the *Adcy4* gene.

We previously demonstrated that AC6 regulates CD Na and water transport (Roos et al. 2012, 2013; Kittikulsuth et al. 2014). While CD AC6 KO abolished AVP‐stimulated ENaC activity, it only modestly reduced urinary concentrating ability and AVP‐induced cAMP accumulation. Further, CD AC3 KO mice had no detectable alterations in urinary Na or water excretion or BP (Kittikulsuth et al. 2014) (although AC3 siRNA did reduce AVP‐stimulated cAMP levels in cultured IMCD cells (Strait et al. 2010), no such effect on AVP‐induced cAMP accumulation was detected in CD AC3 KO mice). Given the modest effect on water handling of CD AC6 KO, and that CD AC3 KO and CD AC4 KO have no evident effect on CD water handling, the possibility is raised that other AC isoform(s) may be involved in CD water transport. We did not detect membrane‐bound AC isoforms in CD principal cells beyond AC3, AC4, and AC6 (Strait et al. 2010); however, it is possible that other membrane‐bound AC isoforms are present. In addition, the soluble AC is expressed in principal cells of the CD and may regulate CD Na and acid/base handling (Hallows et al. 2009; Păunescu et al. 2010). It is possible that the soluble AC is also involved in CD water handling; experiments examining this issue would be interesting. Another important and related consideration is that there may be differences between medullary and cortical CD with regard to AC isoform function. In particular, Rieg et al. (2010) reported that mice with whole animal AC6 KO had markedly reduced AQP2 phosphorylation in the inner medulla, but not in cortex or outer medulla. Finally, while we did not detect differences in AC6 mRNA between CD AC4 KO and control mice, it is possible that upregulation of AC6 protein could have masked an effect of CD AC4 KO. This question could ultimately be addressed by examining the effect of combined CD AC4/AC6 KO on renal Na and water excretion and BP.

The current study has some limitations. First, as it is not possible to measure AC4 directly in principal cells due to the insensitivity of available techniques, one cannot be certain that AC4 is completely eliminated from all principal cells. While a significant reduction in AC4 mRNA was seen in inner medulla and isolated CCD from CD AC4 KO, complete abolition of AC4 mRNA was not observed; this is likely due, at least in part, to the presence of nonprincipal cell types. We did detect complete AC4 gene recombination in isolated IMCD, however, such findings are not seen in isolated CCD due to lack of targeting intercalated cells. While previous studies with AQP2‐Cre mice have shown about 95% recombination in principal cells, one cannot generalize to all floxed alleles. Thus, our results should be interpreted with the caveat that it is impossible to distinguish between knockdown and complete knockout of AC4 expression in principal cells. Second, we examined the in vivo phenotype of CD AC4 KO mice with regard to Na and water handling in detail, however, even more extensive studies could have been conducted, including administration of exogenous AVP in the setting of normal‐ or high‐water intake, analysis of AQP2 phosphorylation, determination of ENaC isoform expression, etc. However, given the absence of even a trend toward a difference in renal Na or water excretion, plasma hormones or BP between CD AC4 KO and control mice, these additional studies seemed highly unlikely to be informative.

In summary, the current study demonstrates that CD AC4 KO mice do not have altered BP or renal Na and water handling under varying physiologic conditions. Whether and how AC4 modulates other aspects of CD, or even whole body function, remains to be determined. In addition, the possible role of other CD AC isoforms, including soluble AC, in modulating CD Na and water transport, requires further elucidation.

## Acknowledgments

The technical assistance of Shuping Wang is appreciated.

## Conflict of Interest

None declared.
